# Construction and validation of a machine learning–based risk prediction model for venous thromboembolism in older adult patients: A multifactorial analysis of 28,231 cases

**DOI:** 10.1097/MD.0000000000049729

**Published:** 2026-07-17

**Authors:** Siyu Zhou, Zhenrui Tang, Yifeng Tan, Nian Ni, Dong Liang

**Affiliations:** a Cardiothoracic Surgery, Bishan Hospital of Chongqing Medical University, Bishan, Chongqing, China.

**Keywords:** diabetic nephropathy, older adults, real-world data, risk prediction, SHAP, venous thromboembolism, XGBoost

## Abstract

Venous thromboembolism (VTE) is a leading preventable cause of in-hospital mortality in older adults, yet early risk stratification remains a key clinical challenge. This study aimed to develop and internally validate an explainable machine learning model for incident VTE prediction in hospitalized older adult patients. We enrolled 28,231 patients aged ≥65 years admitted between January 2023 and December 2024, excluding those with VTE on admission. The primary endpoint was imaging-confirmed incident in-hospital VTE. Patients were split into training/test sets (7:3) via outcome-stratified sampling. Missing data were handled with multivariate imputation by chained equations imputation (training set only). Five machine learning models were constructed with 10-fold cross-validation and hyperparameter tuning, evaluated by pooled area under the curve (AUC), calibration curves, and decision curve analysis, with SHAP for model interpretation. 1797 (6.38%) incident VTE events were recorded. XGBoost showed optimal performance, with a training AUC of 0.753 and a test AUC of 0.712, favorable calibration, and stable clinical net benefit. Top predictors included diabetic nephropathy, triglycerides, great saphenous vein varicosity, fatty liver, cerebrovascular accident and age. We developed and validated an explainable XGBoost model for VTE risk prediction in older inpatients, enabling early risk stratification to support individualized thromboprophylaxis. Multicenter prospective external validation is warranted for clinical implementation.

## 1. Introduction

Venous thromboembolism (VTE), comprising deep vein thrombosis (DVT) and pulmonary embolism, is one of the leading preventable causes of in-hospital death among hospitalized older adult patients and a major global public health concern.^[1]^ The global annual incidence of leg DVT is reported to be approximately 1.6 per 1000 people.^[2]^ With the aging population, rising prevalence of chronic diseases, and increased surgical interventions, the incidence of VTE has been steadily increasing.^[3]^ Currently, the main treatment and secondary prevention of VTE is anticoagulant therapy. However, despite continuous improvements in anticoagulant drugs and preventive strategies, the early identification of high-risk patients among hospitalized older adult patients remains a significant clinical challenge due to the insidious onset of VTE, lack of specific clinical manifestations, and difficulty in early diagnosis.^[1,4]^

Extensive epidemiological evidence has demonstrated that the development of VTE in hospitalized older adult patients is closely associated with various risk factors, including prolonged immobilization, malignancy, cardiovascular diseases, metabolic disorders, inflammatory responses, and invasive procedures.^[5–7]^ These factors contribute synergistically to abnormalities in blood flow, endothelial dysfunction, and hypercoagulability, ultimately triggering the pathological processes represented by Virchow triad. However, traditional statistical approaches such as logistic regression and Cox proportional hazards models face inherent limitations in dealing with complex, multidimensional data characterized by nonlinearities and high-order interactions. These models generally assume independence and linear relationships among predictors, which may oversimplify the intricate mechanisms of thrombogenesis and restrict predictive performance and clinical applicability.^[8]^ Therefore, there is an urgent need for more advanced analytical methodologies capable of seamlessly integrating multisource clinical data, laboratory indicators, and procedure-related variables, enabling a more comprehensive understanding of VTE pathogenesis and achieving more accurate risk stratification and early prediction.

Recent years have seen rapid advancements in machine learning (ML) technologies, bringing new opportunities for predictive modeling in medicine.^[9]^ Compared with traditional statistical methods, ML algorithms are capable of automatically identifying underlying patterns in large-scale, high-dimensional datasets, capturing complex nonlinear relationships and high-order interactions among variables to substantially enhance prediction performance.^[10]^ Their advantages have been demonstrated across diverse medical fields, including cardiovascular event prediction,^[11]^ metabolic disease risk assessment,^[12]^ and thrombotic disease research.^[13]^ Specifically, in cardiovascular and cerebrovascular research, ML enables the integration of clinical characteristics, imaging features, biochemical indicators, and genetic profiles, thereby overcoming the limitations of traditional models that rely on linear assumptions and single-dimension variables. Likewise, for metabolic syndrome and diabetes prediction, ML algorithms have proven effective in identifying nonlinear associations within complex metabolic networks and inflammatory markers, achieving significantly improved accuracy in early disease detection. Leveraging these strengths, ML-based modeling approaches offer new directions for individualized VTE risk stratification, dynamic prediction, and precision prevention in hospitalized older adult patients.^[14]^

Given this context, the present study utilizes a large real-world clinical dataset to systematically identify clinical characteristics, biochemical parameters, and procedural factors closely associated with VTE development and to construct multiple ML prediction models. Five algorithms- multivariable logistic regression, elastic net, random forest, XGBoost, and a neural network- were employed, with 10-fold cross-validation and hyperparameter tuning for model optimization. Model predictive performance and clinical applicability were comprehensively evaluated using receiver operating characteristic (ROC) curves, decision curve analysis (DCA), and calibration plots, while key contributors in the optimal model were interpreted via permutation feature importance and partial dependence–individual conditional expectation (PD–ICE) curves. The aim of this study is to establish a highly accurate, interpretable, and clinically valuable VTE risk prediction model, providing scientific evidence for the early identification and personalized prevention of high-risk individuals in hospitalized older adult patients.

## 2. Materials and methods

### 2.1. Data source

The study was conducted for all patients who were residents of all medical departments of Bishan Hospital of Chongqing Medical University between January 2023 and December 2024, with ethical considerations as outlined by Bishan Hospital of Chongqing (approved number cqbykyll-20240918-16). The data for patients who were ≥65 years of age and had a ≥2-day duration of hospitalization were included. VTE events were defined as incident events occurring after hospital admission, and all cases were confirmed by standardized imaging examinations. Patients who had VTE on admission, were <65 years of age, lacked major indications and experimental data (more than 7 parameters were missing), and had uncertainties in the acquisition time for laboratory indicators were excluded. Extensive clinical information was collected, including patients’ medical histories, surgical interventions, and VTE-related biochemical parameters. The aim was to explore clinical exposures highly associated with the occurrence and progression of VTE and to develop predictive models to support clinical diagnosis and risk assessment. The study variables primarily included: age; myocardial infarction (total) (MI); acute non-ST-elevation myocardial infarction; acute ST-elevation myocardial infarction; old myocardial infarction; myocardial infarction location; peripheral arterial disease (total); great saphenous varicose veins (GSVV); lower limb varicose veins; edema; gastric varices; esophagogastric varices; cerebrovascular disease (total); cerebrovascular stenosis; ischemic cerebrovascular disease; cerebral insufficiency; heart failure (total); acute heart failure; chronic heart failure; hypertensive heart disease; rheumatic diseases (total); rheumatoid arthritis; rheumatic heart disease; immune system diseases (total); purpura; systemic lupus erythematosus; allergic dermatitis; gastrointestinal bleeding (total) (GH); upper gastrointestinal bleeding; lower gastrointestinal bleeding; stress-induced gastrointestinal bleeding; gastrointestinal perforation (GIP); diabetes mellitus (total); type 2 diabetes mellitus; diabetic ketoacidosis; diabetic foot; diabetic nephropathy; nephropathies (total) (NP); renal insufficiency (RI); pyelonephritis; nephritis; nephrotic syndrome; hemiplegia; liver disease (total); hepatic cyst; hepatic hemangioma; fatty liver (FL); intrahepatic bile duct stones; hepatic insufficiency (HI); liver cirrhosis; hepatitis B (HB); pulmonary embolism; DVT of the lower extremities (DVT); cerebrovascular accident (CVA); transient ischemic attack; peripherally inserted central catheter placement; triglycerides; total cholesterol; fibrinogen; hemoglobin; platelets (thrombocyte); high-sensitivity C-reactive protein (CRP); activated partial thromboplastin time (APTT); prothrombin time; white blood cell count; and red blood cell count (RBC).

### 2.2. Overall baseline characteristics and preliminary univariate assessment

Initially, the raw clinical data collected in this study underwent systematic cleaning and standardized preprocessing to ensure data quality and reliability for subsequent analyses. Categorical variables were uniformly standardized as factor type: dichotomous variables were coded using the standard binary assignment of 0 (negative) and 1 (positive), with descriptive statistics presented as “frequency (proportion) [n (%)]”; continuous numerical variables were standardized as numeric type, with initial descriptive statistics expressed as “mean ± standard deviation (x̄ ± s).” During preprocessing, patient samples with missing outcome information were excluded, and baseline features with missing value proportions exceeding 40% were eliminated, yielding a final cohort of 28,231 eligible patient records (Figure S1A, Supplemental Digital Content 1).

All baseline indicators were compared between groups stratified by study outcome (VTE case group vs control group), with appropriate statistical methods selected based on variable type and data distribution characteristics: for continuous variables following normal distribution with homogeneity of variance, the independent two-sample t-test was employed; for continuous variables deviating from normal distribution or with heteroscedasticity, the Mann–Whitney U test was applied; for categorical variables, the *χ*^2^ test was used, with Fisher exact test substituted when total sample size n < 40 or expected frequency *T* < 1 to avoid biased test results.

Furthermore, univariate logistic regression analysis was conducted, with study outcome grouping (VTE case group = 1, control group = 0) as the dependent variable and each baseline characteristic as the independent variable. Separate univariate logistic regression models were constructed to calculate regression coefficients (β), standard errors, odds ratios (OR), and 95% confidence intervals (95% confidence interval [CI]) for each independent variable, along with corresponding *P*-values. An OR > 1 indicated the characteristic as a risk factor for VTE occurrence, OR < 1 suggested a protective factor, and OR = 1 indicated no association with VTE occurrence. This preliminary screening identified potential influencing factors associated with the study outcome, laying the foundation for subsequent feature selection and model construction.

### 2.3. Dataset partitioning, feature selection, and quality control of multiple imputation

Furthermore, eligible subjects were randomly allocated into training and testing sets using stratified sampling at a 7:3 ratio, with study outcome serving as the stratification variable. This approach ensured comparable baseline characteristics and outcome distributions between the 2 cohorts, thereby establishing a robust data foundation for subsequent model development and validation. Within the training cohort, multicollinearity assessment was performed for all baseline features by calculating variance inflation factors (VIFs), and features exhibiting VIF values exceeding 10 were systematically excluded to mitigate redundant interference among predictors. Missing values were subsequently addressed using the multivariate imputation by chained equations algorithm within the training set exclusively, generating ten imputed datasets. Convergence and imputation quality were evaluated by plotting the mean and standard deviation trends of each feature across all imputed datasets (Figure S1B, Supplemental Digital Content 1). To fundamentally prevent data leakage and ensure the integrity of model validation, all procedures, including model training, hyperparameter optimization, and feature selection, were strictly confined to the training set, with the testing set maintained in complete isolation throughout the entire analytical pipeline.

### 2.4. Construction of machine learning prediction models and identification of the optimal model

Based on the 10 multiply imputed training sets, we employed 5 ML algorithms: multivariable logistic regression, elastic net, random forest, XGBoost, and neural network, to explore intrinsic data associations from diverse modeling perspectives and achieve optimal predictive performance for the study outcome. To ensure all algorithms were constructed within a unified and comparable technical framework, the “mlr3” package (version 1.3.0) was utilized as the model development platform.^[15]^ During model construction, 10-fold cross-validation was implemented within the training set to mitigate overfitting risks and maximize data utilization. Hyperparameter tuning for each algorithm was performed using the “mlr3tuning” package (version 1.5.1) with grid search algorithms to identify the optimal parameter combinations for each algorithm within the training set, thereby constructing the corresponding optimal prediction models.

Subsequently, all models developed across the 10 imputed datasets were evaluated using the independent, holdout test set. ROC curve analysis was conducted for each model corresponding to every imputed dataset, and the area under the curve (AUC) was calculated to assess predictive efficacy. Following Rubin Rules, AUC values from the 10 imputed datasets were pooled for each ML algorithm to obtain the corresponding pooled AUC. The difference between the training set pooled AUC and the test set pooled AUC was further calculated to evaluate potential overfitting. Additionally, the coefficient of variation (CV) of pooled AUC across both datasets was computed to quantitatively assess model predictive stability. Ultimately, the algorithm exhibiting the maximal testing set pooled AUC, minimal difference between training and test set pooled AUC, and CV < 5% was selected as the optimal prediction model for this study.

### 2.5. In-depth evaluation and interpretation of the optimal prediction model

To further analyze the selected optimal ML prediction model and elucidate its predictive efficacy, clinical applicability, and feature mechanisms, the “iml” package (version 0.11.4) was employed to generate confusion matrices, DCA curves, calibration curves, and partial dependence-individual conditional expectation (PD-ICE) curves for both the training and test sets. Specifically, confusion matrices were constructed to visually present the concordance between predicted and actual outcomes; DCA curves were utilized to evaluate the clinical net benefit; calibration curves were employed to validate the agreement between predicted probabilities and observed outcome frequencies; and PD-ICE curves were generated to illustrate the marginal effects of individual features on the outcome and their inter-individual variability.

Concurrently, SHAP (SHapley Additive exPlanations) analysis was conducted to calculate SHAP values (φ values) for each feature across the 10 multivariate imputation by chained equations-imputed datasets within the optimal model. These feature-specific SHAP values were subsequently pooled using Rubin Rules to objectively quantify the contribution magnitude and direction of each feature to model predictions, thereby clarifying the potential associations and relative strengths between individual features and the study outcome.

Furthermore, comprehensive performance metrics including accuracy, precision, recall, F1-score, and specificity were calculated for the optimal model in both the training and test sets. These multidimensional evaluations provided a thorough assessment of predictive validity, robustness, and generalizability, offering reliable evidence to support the clinical application of the model.

## 3. Results

### 3.1. Baseline characteristics and univariate logistic regression analysis results

A total of 28,231 eligible subjects were enrolled in this study and stratified according to study outcome into the control group (n = 26,434) and the case group (n = 1797). Baseline characteristics and univariate logistic regression analysis results for both groups are presented in Table 1.

**Table 1:** Clinical characteristics of patients stratified by VTE status and univariate logistic regression results.

	Percentage (%) or mean ± standard deviation		*P*	OR	P.ratio
	Control (N = 26,434)	VTE (N = 1797)			
Age	75.4 (7.10)	77.0 (7.15)	<.001	1.031 (1.025; 1.038 (	<0.001
Non-ST elevation myocardial infarction:			.265		
No	26,302 (99.5%)	1784 (99.3%)		Ref.	Ref.
Yes	132 (0.50%)	13 (0.72%)		1.468 (0.787; 2.502)	0.212
ST elevation myocardial infarction:			.038		
No	26,370 (99.8%)	1797 (100%)		Ref.	Ref.
Yes	64 (0.24%)	0 (0.00%)		(.;)	
Old myocardial infarction:			1.000		
No	26,403 (99.9%)	1795 (99.9%)		Ref.	Ref.
Yes	31 (0.12%)	2 (0.11%)		1.018 (0.153; 3.367)	0.981
Great saphenous varicose veins:			<.001		
No	26,329 (99.6%)	1774 (98.7%)		Ref.	Ref.
Yes	105 (0.40%)	23 (1.28%)		3.269 (2.025; 5.051)	<0.001
Lower limb Varicose veins:			.103		
No	26,343 (99.7%)	1786 (99.4%)		Ref.	Ref.
Yes	91 (0.34%)	11 (0.61%)		1.806 (0.907; 3.237)	0.088
Small saphenous varicose veins:			1.000		
No	26,431 (100.0%)	1797 (100%)		Ref.	Ref.
Yes	3 (0.01%)	0 (0.00%)		(.;.)	
Edema:			<.001		
No	26,275 (99.4%)	1770 (98.5%)		Ref.	Ref.
Yes	159 (0.60%)	27 (1.50%)		2.533 (1.643; 3.755)	<0.001
Gastric varices:			0.781		
No	26,385 (99.8%)	1793 (99.8%)		Ref.	Ref.
Yes	49 (0.19%)	4 (0.22%)		1.245 (0.368; 3.063)	0.685
Esophageal and gastric varices:			1.000		
No	26,412 (99.9%)	1796 (99.9%)		Ref.	Ref.
Yes	22 (0.08%)	1 (0.06%)		0.761 (0.032; 3.595)	0.785
Acute cerebrovascular disease:			.326		
No	26,429 (100.0%)	1796 (99.9%)		Ref.	Ref.
Yes	5 (0.02%)	1 (0.06%)		3.269 (0.124; 21.265)	0.377
Cerebral vascular stenosis:			<.001		
No	25,694 (97.2%)	1785 (99.3%)		Ref.	Ref.
Yes	740 (2.80%)	12 (0.67%)		0.237 (0.126; 0.400)	<0.001
Cerebral aneurysm:			1.000		
No	26,431 (100.0%)	1797 (100%)		Ref.	Ref.
Yes	3 (0.01%)	0 (0.00%)		(.;.)	
Ischemic cerebrovascular disease:			.179		
No	26,396 (99.9%)	1797 (100%)		Ref.	Ref.
Yes	38 (0.14%)	0 (0.00%)		(.;.)	
Cerebral vascular malformation:			1.000		
No	26,427 (100.0%)	1797 (100%)		Ref.	Ref.
Yes	7 (0.03%)	0 (0.00%)		(.;.)	
Cerebral ischemia:			1.000		
No	26,405 (99.9%)	1795 (99.9%)		Ref.	Ref.
Yes	29 (0.11%)	2 (0.11%)		1.087 (0.163; 3.618)	0.911
Acute heart failure:			.464		
No	26,062 (98.6%)	1776 (98.8%)		Ref.	Ref.
Yes	372 (1.41%)	21 (1.17%)		0.834 (0.519; 1.265)	0.411
Chronic heart failure:			.036		
No	26,023 (98.4%)	1757 (97.8%)		Ref.	Ref.
Yes	411 (1.55%)	40 (2.23%)		1.447 (1.026; 1.983)	0.036
Hypertensive heart disease with heart failure:			.029		
No	26,317 (99.6%)	1782 (99.2%)		Ref.	Ref.
Yes	117 (0.44%)	15 (0.83%)		1.911 (1.067; 3.174)	0.031
Refractory heart failure:			.053		
No	26,430 (100.0%)	1795 (99.9%)		Ref.	Ref.
Yes	4 (0.02%)	2 (0.11%)		7.629 (0.942; 41.545)	0.056
Diastolic heart failure:			1.000		
No	26,428 (100.0%)	1797 (100%)		Ref.	Ref.
Yes	6 (0.02%)	0 (0.00%)		(.;.)	
Rheumatoid arthritis:			1.000		
No	26,221 (99.2%)	1782 (99.2%)		Ref.	Ref.
Yes	213 (0.81%)	15 (0.83%)		1.047 (0.591; 1.709)	0.866
Rheumatic heart disease:			.318		
No	26,309 (99.5%)	1792 (99.7%)		Ref.	Ref.
Yes	125 (0.47%)	5 (0.28%)		0.605 (0.212; 1.334)	0.236
Purpura:			1.000		
No	26,424 (100.0%)	1796 (99.9%)		Ref.	Ref.
Yes	10 (0.04%)	1 (0.06%)		1.660 (0.067; 8.777)	0.667
Systemic lupus erythematosus:			.415		
No	26,418 (99.9%)	1797 (100%)		Ref.	Ref.
Yes	16 (0.06%)	0 (0.00%)		(.;.)	
Allergic dermatitis:			.068		
No	26,404 (99.9%)	1792 (99.7%)		Ref.	Ref.
Yes	30 (0.11%)	5 (0.28%)		2.521 (0.845; 5.985)	0.091
Upper gastrointestinal bleeding:			<.001		
No	26,129 (98.8%)	1749 (97.3%)		Ref.	Ref.
Yes	305 (1.15%)	48 (2.67%)		2.358 (1.712; 3.177)	<0.001
Lower gastrointestinal bleeding:			1.000		
No	26,408 (99.9%)	1795 (99.9%)		Ref.	Ref.
Yes	26 (0.10%)	2 (0.11%)		1.212 (0.181; 4.070)	0.802
Stress-induced gastrointestinal bleeding:			<.001		
No	26,404 (99.9%)	1786 (99.4%)		Ref.	Ref.
Yes	30 (0.11%)	11 (0.61%)		5.471 (2.603; 10.653)	<0.001
Gastrointestinal perforation:			<.001		
No	26,367 (99.7%)	1780 (99.1%)		Ref.	Ref.
Yes	67 (0.25%)	17 (0.95%)		3.785 (2.144; 6.312)	<0.001
Type 1 diabetes mellitus:			1.000		
No	26,427 (100.0%)	1797 (100%)		Ref.	Ref.
Yes	7 (0.03%)	0 (0.00%)		(.;.)	
Type 2 diabetes mellitus:			<.001		
No	20,463 (77.4%)	1472 (81.9%)		Ref.	Ref.
Yes	5971 (22.6%)	325 (18.1%)		0.757 (0.668; 0.855)	<0.001
Diabetic ketoacidosis:			.506		
No	26,243 (99.3%)	1787 (99.4%)		Ref.	Ref.
Yes	191 (0.72%)	10 (0.56%)		0.781 (0.384; 1.400)	0.431
Diabetic peripheral neuropathy:			0.668		
No	26,426 (100.0%)	1797 (100%)		Ref.	Ref.
Yes	8 (0.03%)	0 (0.00%)		(.;.)	
Diabetic foot:			1.000		
No	26,392 (99.8%)	1794 (99.8%)		Ref.	Ref.
Yes	42 (0.16%)	3 (0.17%)		1.102 (0.258; 3.034)	0.874
Diabetic nephropathy:			.013		
No	26,208 (99.1%)	1792 (99.7%)		Ref.	Ref.
Yes	226 (0.85%)	5 (0.28%)		0.334 (0.117; 0.728)	0.003
Nephropathy:			<.001		
No	24,230 (91.7%)	1584 (88.1%)		Ref.	Ref.
Yes	2204 (8.34%)	213 (11.9%)		1.479 (1.271; 1.714)	<0.001
Renal insufficiency:			<.001		
No	24,407 (92.3%)	1592 (88.6%)		Ref.	Ref.
Yes	2027 (7.67%)	205 (11.4%)		1.551 (1.329; 1.802)	<0.001
Pyelonephritis:			.807		
No	26,376 (99.8%)	1794 (99.8%)		Ref.	Ref.
Yes	58 (0.22%)	3 (0.17%)		0.799 (0.188; 2.161)	0.697
Nephritis:			.586		
No	26,386 (99.8%)	1795 (99.9%)		Ref.	Ref.
Yes	48 (0.18%)	2 (0.11%)		0.658 (0.100; 2.120)	0.539
Nephrotic syndrome:			.826		
No	26,366 (99.7%)	1793 (99.8%)		Ref.	Ref.
Yes	68 (0.26%)	4 (0.22%)		0.898 (0.268; 2.173)	0.832
Hemiplegia:			.352		
No	26,393 (99.8%)	1796 (99.9%)		Ref.	Ref.
Yes	41 (0.16%)	1 (0.06%)		0.409 (0.018; 1.853)	0.306
Hepatic cirrhosis:			.311		
No	25,813 (97.7%)	1762 (98.1%)		Ref.	Ref.
Yes	621 (2.35%)	35 (1.95%)		0.829 (0.577; 1.151)	0.273
Hepatic hemangioma:			.457		
No	26,339 (99.6%)	1793 (99.8%)		Ref.	Ref.
Yes	95 (0.36%)	4 (0.22%)		0.642 (0.193; 1.537)	0.355
Fatty liver:			.001		
No	25,818 (97.7%)	1777 (98.9%)		Ref.	Ref.
Yes	616 (2.33%)	20 (1.11%)		0.475 (0.294; 0.723)	<0.001
Intrahepatic biliary stones:			.518		
No	26,401 (99.9%)	1796 (99.9%)		Ref.	Ref.
Yes	33 (0.12%)	1 (0.06%)		0.508 (0.022; 2.328)	0.460
Hepatic insufficiency:			<.001		
No	25,213 (95.4%)	1662 (92.5%)		Ref.	Ref.
Yes	1221 (4.62%)	135 (7.51%)		1.679 (1.390; 2.012)	<0.001
Liver cirrhosis:			.184		
No	26,272 (99.4%)	1791 (99.7%)		Ref.	Ref.
Yes	162 (0.61%)	6 (0.33%)		0.557 (0.217; 1.154)	0.124
Hepatitis B:			.926		
No	26,212 (99.2%)	1781 (99.1%)		Ref.	Ref.
Yes	222 (0.84%)	16 (0.89%)		1.071 (0.617; 1.724)	0.795
Pulmonary embolism:			<.001		
No	26,434 (100%)	1135 (63.2%)		Ref.	Ref.
Yes	0 (0.00%)	662 (36.8%)		(.;.)	
Deep vein thrombosis of the lower limbs:			<.001		
No	26,434 (100%)	558 (31.1%)		Ref.	Ref.
Yes	0 (0.00%)	1239 (68.9%)		(.;.)	
Cerebrovascular accident:			.001		
No	25,891 (97.9%)	1739 (96.8%)		Ref.	Ref.
Yes	543 (2.05%)	58 (3.23%)		1.594 (1.198; 2.080)	0.002
Transient ischemic attack:			.423		
No	26,319 (99.6%)	1792 (99.7%)		Ref.	Ref.
Yes	115 (0.44%)	5 (0.28%)		0.658 (0.230; 1.453)	0.331
Peripherally inserted central catheter:			.641		
No	26,413 (99.9%)	1795 (99.9%)		Ref.	Ref.
Yes	21 (0.08%)	2 (0.11%)		1.499 (0.222; 5.140)	0.613
Triglyceride	1.70 (1.24)	1.47 (0.92)	<.001	0.797 (0.748; 0.849)	<0.001
Total cholesterol	4.29 (1.18)	4.21 (1.20)	.017	0.944 (0.901; 0.989)	0.015
Fibrinogen	4.00 (1.75)	4.22 (2.01)	<.001	1.065 (1.038; 1.091)	<0.001
Hemoglobin	121 (21.2)	114 (22.6)	<.001	0.985 (0.983; 0.987)	<0.001
Thrombocyte (Platelet)	207 (86.7)	207 (88.9)	.954	1.000 (0.999; 1.001)	0.953
C-reactive protein	32.4 (47.4)	50.0 (59.9)	<.001	1.006 (1.005; 1.007)	<0.001
Activated partial thromboplastin Time	25.8 (3.68)	26.8 (5.10)	<.001	1.044 (1.034; 1.055)	<0.001
Prothrombin time	10.8 (2.05)	11.7 (3.57)	<.001	1.106 (1.088; 1.125)	<0.001
White blood cell	7.24 (4.08)	8.03 (4.38)	<.001	1.032 (1.022; 1.041)	<0.001
Red blood cell	4.06 (0.70)	3.81 (0.75)	<.001	0.619 (0.580; 0.662)	<0.001

Among continuous variables, the mean ages in the control and case groups were 75.4 ± 7.10 years and 77.0 ± 7.15 years, respectively. Univariate logistic regression analysis revealed a significant association between age and study outcome (OR = 1.03, 95% CI: 1.02–1.04, *P* < .001). Regarding lipid-related indicators, triglyceride and total cholesterol levels in the control group were 1.70 ± 1.24 and 4.29 ± 1.18, respectively, compared to 1.47 ± 0.92 and 4.21 ± 1.20 in the case group. Both triglycerides (OR = 0.80, 95% CI: 0.75–0.85, *P* < .001) and total cholesterol (OR = 0.94, 95% CI: 0.90–0.99, *P* = .015) demonstrated significant associations with the outcome. For coagulation and inflammation-related parameters, fibrinogen, CRP, APTT, and prothrombin time were all elevated in the case group compared to the control group, exhibiting significant positive correlations with the outcome (all *P* < .001). Conversely, RBC was lower in the case group (3.81 ± 0.75) than in the control group (4.06 ± 0.70), showing a significant negative association (OR = 0.62, 95% CI: 0.58–0.66, *P* < .001).

Among categorical variables, statistically significant differences in distribution between the 2 groups were observed for great saphenous vein varicosity, edema, cerebral vascular stenosis, upper GH, stress-related GH, GIP, type 2 diabetes mellitus, nephropathy, RI, HI, and CVA (all *P* < .05). Specifically, great saphenous vein varicosity (OR = 3.27, 95% CI: 2.02–5.05, *P* < .001), stress-related GH (OR = 5.47, 95% CI: 2.60–10.7, *P* < .001), and GIP (OR = 3.79, 95% CI: 2.14–6.31, *P* < .001) were identified as risk factors for the outcome. In contrast, non-ST-segment elevation MI, old MI, gastric varices, and acute cerebrovascular disease showed no statistically significant differences in distribution between groups (all *P* > .05), with univariate logistic regression analysis indicating no significant associations with the study outcome.

**Figure 1.**
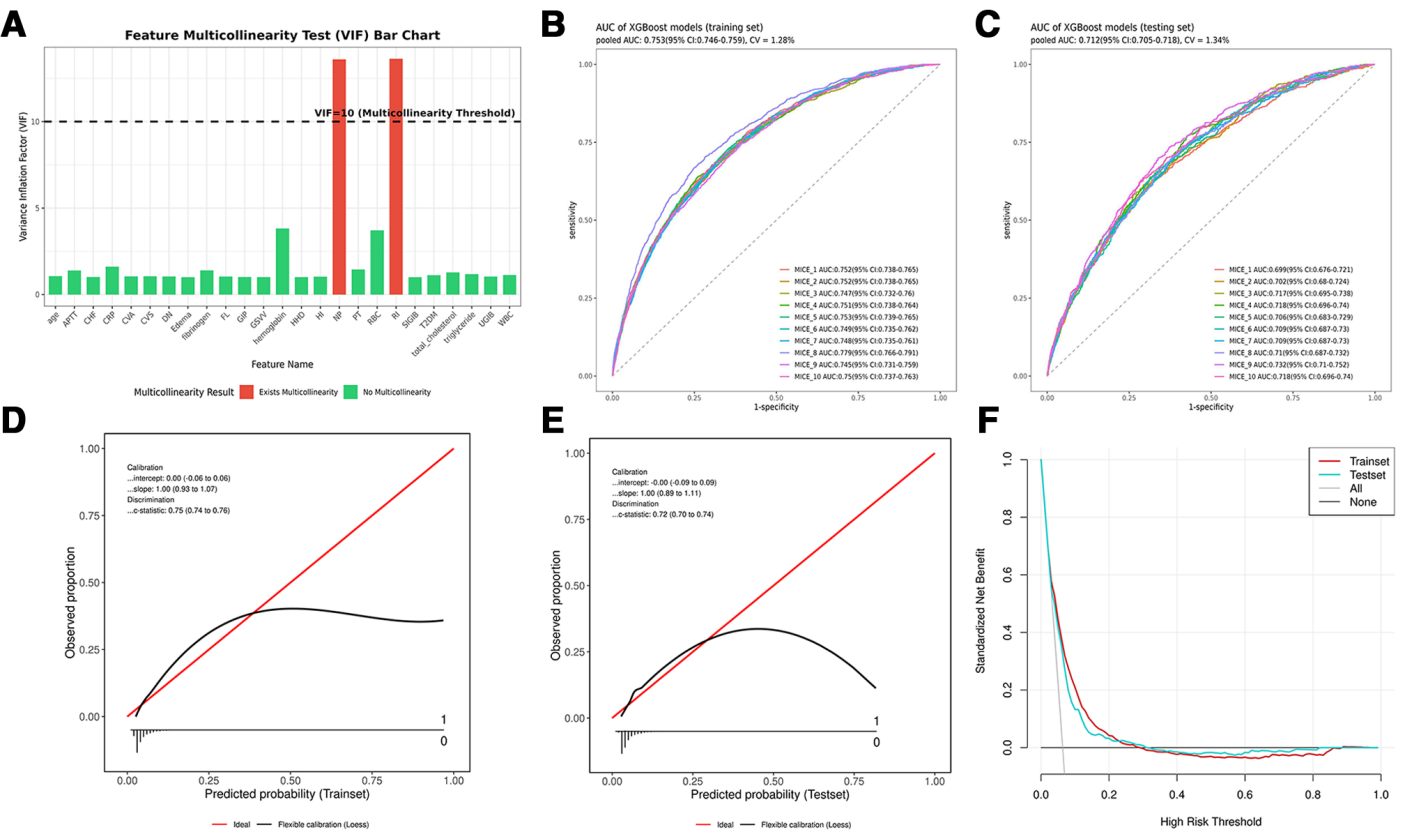
Performance evaluation of the optimal XGBoost prediction model. (A) Variance inflation factor (VIF) analysis for feature multicollinearity assessment; (B) Pooled AUC values of 5 machine learning models in the training set; (C) Pooled AUC values of 5 machine learning models in the test set; (D) Calibration curve in the training set; (E) Calibration curve in the test set; (F) Decision curve analysis (DCA) in training and test sets. AUC = area under the curve.

Further multicollinearity diagnostics revealed that the vast majority of features exhibited VIF values below 10, indicating the absence of severe multicollinearity and permitting direct inclusion in subsequent model construction. Only 2 features (NP and RI) demonstrated VIF values substantially exceeding 10 (red bars), indicative of severe multicollinearity necessitating exclusion from the feature set. Remaining features, including age, APTT, CRP, and fibrinogen, displayed VIF values at low levels, demonstrating favorable independence among features (Fig. 1A).

### 3.2. Machine learning model construction and identification of the optimal model

In this study, 5 ML models- multivariable logistic regression, elastic net, random forest, XGBoost, and neural network- were constructed based on 10 multiply imputed training sets, with the optimal algorithm selected through comparative evaluation of model performance. Model efficacy was assessed primarily using the pooled area under the ROC curve (pooled AUC), complemented by the AUC difference between training and test sets and the CV for comprehensive evaluation. The results demonstrated that the XGBoost model achieved a pooled AUC of 0.753 (95% CI: 0.746–0.759) in the training set and 0.712 (95% CI: 0.705–0.718) in the test set. Notably, this algorithm exhibited the highest testing set AUC among all candidates, with a minimal AUC difference of 0.041 between training and test sets. Furthermore, the CV values were 1.28% for the training set and 1.34% for the test set, both substantially below the 5% threshold, thereby satisfying the criteria for optimal model selection. Consequently, XGBoost was designated as the optimal prediction model for this study (Fig. 1B, C; Fig. S2 A–H, Supplemental Digital Content 2).

For the training set, the calibration curve intercept was 0.00 (95% CI: −0.06 to 0.06) and the slope was 1.00 (95% CI: 0.93 to 1.07), both approximating ideal values. In the independent validation set, the calibration curve demonstrated substantial consistency with that of the training set, indicating robust generalizability and confirming calibration stability. It is noteworthy that the calibration curves exhibited a characteristic S-shaped pattern, which is consistent with the typical calibration behavior of gradient boosting tree models such as XGBoost (Fig. 1D, E). DCA was employed to evaluate the clinical net benefit of the model across varying risk thresholds (Fig. 1F). The results revealed that standardized net benefit in both training and validation sets significantly exceeded the 2 extreme strategies of “treat all” and “treat none” across a broad threshold range from 0 to approximately 0.8. Within the clinically relevant threshold interval of 0.1 to 0.7, net benefit remained stable and pronounced, indicating that risk stratification using this model can effectively avoid unnecessary interventions or missed diagnoses, thereby demonstrating clear clinical utility. The near-complete overlap of DCA curves between training and validation sets further substantiated the robustness and generalizability of the model.

Additionally, comprehensive performance metrics of the optimal model, including accuracy, precision, recall, F1-score, and specificity, in both training and validation sets are presented in Table 2. The results indicated highly consistent evaluation outcomes between training and test sets, suggesting that the model exhibited no substantial performance degradation on unseen data, maintained stable identification capability for positive events, and possessed reliable generalizability in independent populations. It is noteworthy that precision was relatively low, which is consistent with the highly imbalanced scenario characterized by a positive outcome prevalence of merely approximately 6%. Combined with recall and specificity values around 0.3, this indicates that the model maintains a certain capacity for positive identification while controlling the false positive rate at an acceptable level.

**Table 2:** Performance metrics of the optimal XGBoost model in training and validation Sets.

	Train set					Test set				
	Pooled_value	ci_lower	ci_upper	sd	cv	Pooled_value	ci_lower	ci_upper	sd	cv
Accuracy	0.325485829959514	0.269266194331984	0.395196103238866	0.044308896403198	13.6131568027737	0.353889741470901	0.294132924093968	0.410869436902373	0.0380458686064513	10.7507689961054
Precision	0.0291398668027525	0.0251225116285107	0.0319881851017134	0.00248970755407089	8.54399085254472	0.0324249186675224	0.0268788004408495	0.0363003808612411	0.0031768882331142	9.79767525614873
Recall	0.298647573587908	0.226034208432777	0.357219570405728	0.0450140675125079	15.0726379497129	0.318518518518519	0.237962962962963	0.380648148148148	0.0505494878705512	15.8701880523824
F1	0.0530763423881014	0.0453527590389581	0.0587029876903361	0.00483053971345147	9.10111642232223	0.0588364916732709	0.0482948979174737	0.0662544878264297	0.00607859644737228	10.3313373630905
Specificity	0.327309085013241	0.263935578014376	0.406688104631681	0.0501573860876313	15.3241655622245	0.356298070861178	0.288381036439289	0.422528684907326	0.043839496986388	12.3041634439466

### 3.3. Interpretability analysis of the optimal model

**Figure 2.**
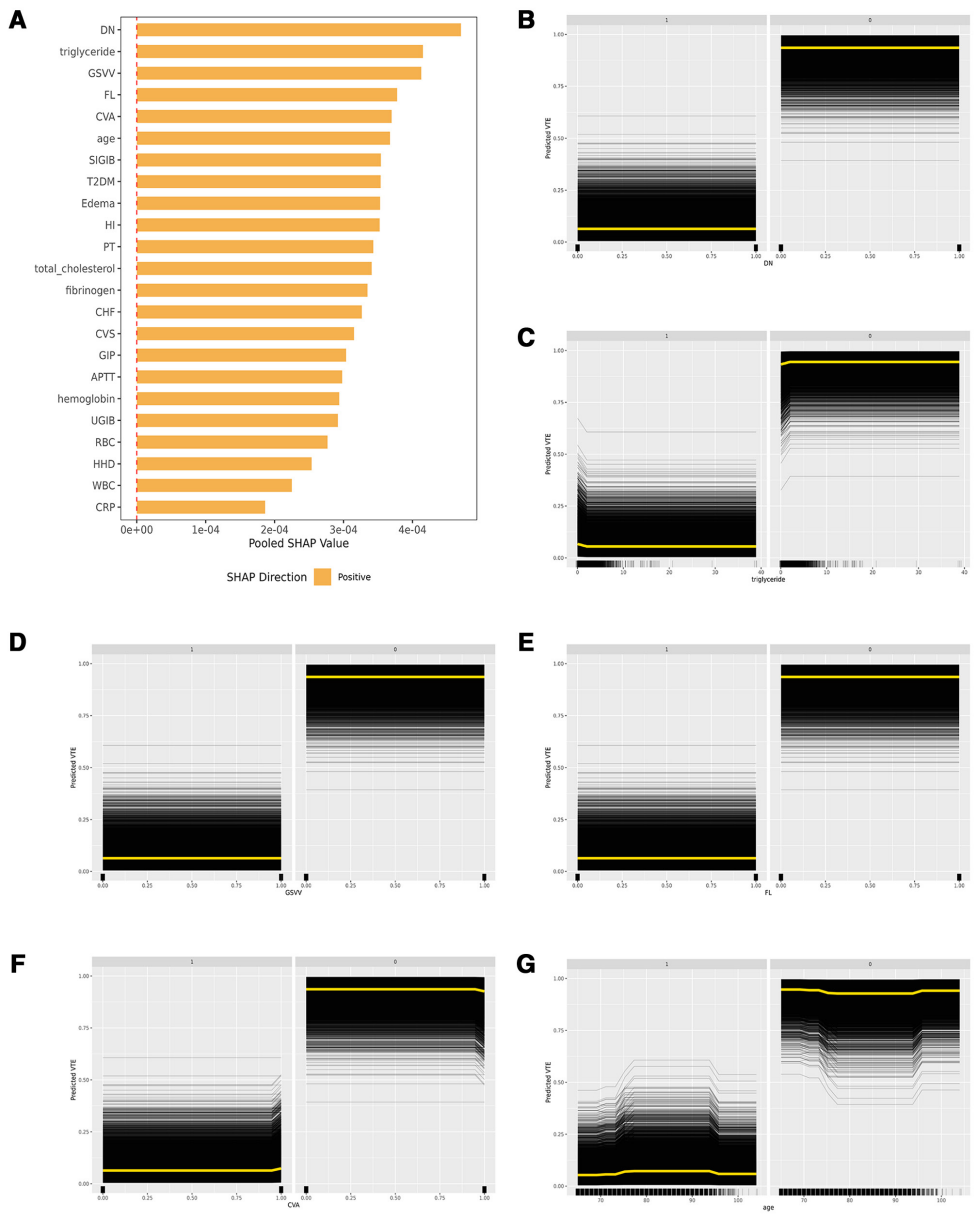
Interpretability analysis of the optimal XGBoost model using SHAP and PD-ICE plots. (A) Pooled SHAP value ranking of all predictive features; (B) PD-ICE plot for diabetic nephropathy (DN); (C) PD-ICE plot for great saphenous vein varicosity (GSVV); (D) PD-ICE plot for fatty liver (FL); (E) PD-ICE plot for cerebrovascular accident (CVA); (F) PD-ICE plot for triglyceride;(G) PD-ICE plot for age. SHAP = SHapley Additive exPlanations.

To elucidate the operational mechanisms of the optimal model, this study aggregated SHAP values across all 10 XGBoost models and evaluated feature contributions to model predictions using pooled SHAP values (Fig 2A). The results revealed substantial heterogeneity in the contributions of different clinical features to outcome prediction. DN, triglyceride, and GSVV exhibited the highest SHAP values, identifying them as the 3 core features driving model predictions and suggesting their pivotal roles in outcome risk stratification. Closely following were FL, CVA, and age, which also demonstrated considerable influence on model outputs.

Further PD-ICE analysis revealed that DN, GSVV, and FL exhibited similar patterns: positive status corresponded to markedly elevated predicted probabilities, whereas negative status was associated with baseline risk, with limited inter-individual heterogeneity. This indicates that these binary features exert a distinct “switch-like” effect on model predictions (Fig. 2B-D). For CVA, predicted probability demonstrated a pronounced stepwise increase, with ICE curves showing a consistent upward shift across individuals, suggesting that CVA, as a well-defined comorbidity, exerts a stable positive driving force on outcome risk (Fig. 2E).

Additionally, as triglyceride levels increased, the model-predicted outcome risk demonstrated a gradual upward trend (Fig. 2F). The influence of age on predicted probability exhibited a nonlinear characteristic: risk increased slowly with advancing age in the 70 to 80 years interval, plateaued in the 80 to 90 years interval, and showed a slight decline beyond 90 years (Fig. 2G).

## 4. Discussion

In this large real-world cohort of 28,231 hospitalized older adults, we developed and validated multiple ML models to predict the risk of VTE. Among the 5 candidate algorithms, XGBoost demonstrated the most favorable overall performance, achieving moderate-to-good discrimination (pooled test AUC 0.712) with minimal performance attenuation between the training and validation sets and low variability across imputed datasets. Importantly, the model exhibited stable calibration and consistent net clinical benefit across a broad range of decision thresholds. Rather than pursuing maximal discrimination alone, this study emphasizes robustness, interpretability, and real-world clinical applicability. The integration of multiple imputation, strict data leakage control, SHAP-based interpretability, and PD–ICE visualization strengthens the reliability and transparency of the predictive framework.

SHAP analysis identified 6 key predictors: DN, TG, GSVV, FL, CVA, and ageas the primary drivers of model prediction. Collectively, these features reflect 3 interrelated pathophysiological axes: metabolic dysfunction, venous structural abnormalities, and systemic vascular injury, superimposed on age-related vulnerability. DN emerged as the most influential predictor. As an advanced microvascular complication of diabetes, DN represents systemic endothelial dysfunction, chronic inflammation, and coagulation–fibrinolysis imbalance.^[16,17]^ Renal impairment is associated with increased fibrinogen levels, platelet activation, and reduced endogenous anticoagulant activity, thereby predisposing patients to a hypercoagulable state.^[18]^ Triglycerides and FL further highlight the role of metabolic dysregulation. Elevated TG levels may indicate underlying metabolic syndrome and contribute to endothelial activation, oxidative stress, and pro-inflammatory cytokine release.^[19]^ FL, increasingly recognized as a systemic inflammatory condition rather than a purely hepatic disorder, has been associated with altered coagulation factor synthesis and impaired fibrinolysis.^[20,21]^ Together, these metabolic factors suggest that VTE risk in older adults may be partially driven by a chronic low-grade inflammatory and prothrombotic milieu. GSVV directly represents venous structural dysfunction and contributes to venous stasis: one of the central components of Virchow triad.^[22]^ Valvular insufficiency and venous dilation promote blood pooling and local coagulation activation.^[23]^ Meanwhile, CVA reflects systemic vascular injury and may indirectly increase VTE risk through reduced mobility, endothelial dysfunction, and prothrombotic alterations following cerebrovascular events.^[24,25]^

Age demonstrated a nonlinear association with predicted VTE risk, with gradual risk elevation in the 70 to 80 year range, plateauing thereafter, and slight decline beyond 90 years. This pattern may reflect threshold effects, survivor bias, or competing mortality risks in the oldest-old population, underscoring the complex interaction between aging biology and thrombosis susceptibility.^[26]^

In contrast, RBC levels were inversely associated with VTE risk in univariate analysis. However, this finding should be interpreted with caution. From a model interpretability perspective, RBC demonstrated relatively low SHAP values compared with the 6 core predictors, indicating limited influence on overall predictive performance. As a retrospective observational study, residual confounding cannot be excluded. RBC levels may reflect underlying anemia, chronic inflammation, frailty, occult malignancy, fluid imbalance, or recent transfusion exposure, all of which may independently affect VTE risk.^[27,28]^ Although physiological hypotheses suggest that RBCs may influence thrombogenesis through modulation of blood rheology and endothelial shear stress, the present analytical framework does not allow causal inference. Therefore, RBC should be interpreted as a noncausal prognostic marker associated with VTE risk rather than a modifiable protective factor. Future prospective and multicenter studies are required to clarify its independent role after adjusting for relevant confounders.”

The integration of metabolic, vascular, and structural venous factors suggests that VTE risk stratification in hospitalized older adults should extend beyond traditional immobilization-based assessment tools. ML-based models incorporating multidimensional clinical data may serve as adjunctive tools for early identification of high-risk individuals, facilitating individualized thromboprophylaxis strategies.

This study has several limitations. First, as a single-center retrospective analysis, selection bias and residual confounding cannot be entirely excluded. Second, external validation in independent populations was not performed. Third, dynamic temporal changes in laboratory parameters were not incorporated. Future multicenter prospective studies are warranted to confirm and refine the model.

## 5. Conclusion

Using a large real-world cohort of hospitalized older adults, we developed and internally validated an explainable ML-based model for VTE risk prediction. The XGBoost model achieved stable discrimination (pooled test AUC 0.712), good calibration, and consistent net clinical benefit across clinically relevant thresholds. Key predictors included diabetic nephropathy, triglycerides, great saphenous vein varicosity, FL, CVA, and age. RBC was inversely associated with VTE in univariate analysis but contributed minimally to the optimal model and should be interpreted as a noncausal prognostic marker. External validation and prospective multicenter studies are warranted before clinical deployment.

## Acknowledgments

We gratefully acknowledge the Bishan Hospital of Chongqing Medical University for the clinical data support.

## Author contributions

**Conceptualization:** Siyu Zhou, Dong Liang.

**Methodology:** Siyu Zhou, Zhenrui Tang.

**Project administration:** Siyu Zhou, Dong Liang.

**Software:** Siyu Zhou.

**Visualization:** Siyu Zhou.

**Formal analysis:** Zhenrui Tang, Dong Liang.

**Resources:** Zhenrui Tang, Yifeng Tan.

**Validation:** Zhenrui Tang, Yifeng Tan.

**Investigation:** Yifeng Tan, Nian Ni.

**Data curation:** Nian Ni.

**Supervision:** Dong Liang.

**Writing – original draft:** Siyu Zhou, Zhenrui Tang, Yifeng Tan, Nian Ni, Dong Liang.

**Writing – review & editing:** Siyu Zhou, Zhenrui Tang, Yifeng Tan, Nian Ni, Dong Liang.





## References

[1] Cohen AT Agnelli G Anderson FA; VTE Impact Assessment Group in Europe (VITAE). Venous thromboembolism (VTE) in Europe. The number of VTE events and associated morbidity and mortality. Thromb Haemost. 2007;98:756–64. 17938798 10.1160/TH07-03-0212

[2] Strijkers RHW Cate-Hoek AJT Bukkems SFFW Wittens CHA. Management of deep vein thrombosis and prevention of post-thrombotic syndrome. BMJ. 2011;343:d5916. 22042752 10.1136/bmj.d5916

[3] Stein PD Hull RD Kayali F Ghali WA Alshab AK Olson RE. Venous thromboembolism according to age: the impact of an aging population. Arch Intern Med. 2004;164:2260–5. 15534164 10.1001/archinte.164.20.2260

[4] Yamashita Y Morimoto T Kimura T. Venous thromboembolism: recent advancement and future perspective. J Cardiol. 2021;79:79–89. 34518074 10.1016/j.jjcc.2021.08.026

[5] Pastori D Cormaci VM Marucci S. A comprehensive review of risk factors for venous thromboembolism: from epidemiology to pathophysiology. Int J Mol Sci . 2023;24:3169. 36834580 10.3390/ijms24043169 PMC9964264

[6] Lutsey PL Zakai NA. Epidemiology and prevention of venous thromboembolism. Nat Rev Cardiol. 2022;20:248–62. 36258120 10.1038/s41569-022-00787-6 PMC9579604

[7] Goldhaber SZ. Risk factors for venous thromboembolism. J Am Coll Cardiol. 2010;56:1–7. 20620709 10.1016/j.jacc.2010.01.057

[8] Chiasakul T Lam BD McNichol M. Artificial intelligence in the prediction of venous thromboembolism: a systematic review and pooled analysis. Eur J Haematol. 2023;111:951–62. 37794526 10.1111/ejh.14110 PMC10900245

[9] Deo RC. Machine learning in medicine. Circulation. 2015;132:1920–30. 26572668 10.1161/CIRCULATIONAHA.115.001593 PMC5831252

[10] Hou T Qiao W Song S. The use of machine learning techniques to predict deep vein thrombosis in rehabilitation inpatients. Clin Appl Thromb Hemost. 2023;29:10760296231179438. 37365805 10.1177/10760296231179438 PMC10326461

[11] Mathur P Srivastava S Xu X Mehta JL. Artificial intelligence, machine learning, and cardiovascular disease. Clin Med Insights Cardiol. 2020;14:1179546820927404. 32952403 10.1177/1179546820927404 PMC7485162

[12] Sghaireen MG Al-Smadi Y Al-Qerem A. Machine learning approach for metabolic syndrome diagnosis using explainable data-augmentation-based classification. Diagnostics (Basel). 2022;12:3117. 36553124 10.3390/diagnostics12123117 PMC9777696

[13] Chen R Petrazzini BO Malick WA Rosenson RS Do R. Prediction of venous thromboembolism in diverse populations using machine learning and structured electronic health records. Arterioscler Thromb Vasc Biol. 2023;44:491–504. 38095106 10.1161/ATVBAHA.123.320331 PMC10872966

[14] Danilatou V Dimopoulos D Kostoulas T Douketis J. Machine learning-based predictive models for patients with venous thromboembolism: a systematic review. Thromb Haemost. 2024;124:1040–52. 38574756 10.1055/a-2299-4758

[15] Qi X Wang S Fang C Jia J Lin L Yuan T. Machine learning and SHAP value interpretation for predicting comorbidity of cardiovascular disease and cancer with dietary antioxidants. Redox Biol. 2024;79:103470. 39700695 10.1016/j.redox.2024.103470 PMC11729017

[16] Alsharidah AS. Diabetes mellitus and diabetic nephropathy: a review of the literature on hemostatic changes in coagulation and thrombosis. Blood Res. 2022;57:101–5. 10.5045/br.2022.2021204 PMC9242838 35620906

[17] Rustiasari UJ Roelofs JJ. The role of platelets in diabetic kidney disease. Int J Mol Sci . 2022;23:8270. 35955405 10.3390/ijms23158270 PMC9368651

[18] Yang J Liu Z. Mechanistic pathogenesis of endothelial dysfunction in diabetic nephropathy and retinopathy. Front Endocrinol (Lausanne). 2022;13:816400. 35692405 10.3389/fendo.2022.816400 PMC9174994

[19] Hamooya BM Siame L Muchaili L Masenga SK Kirabo A. Metabolic syndrome: epidemiology, mechanisms, and current therapeutic approaches. Front Nutr. 2025;12:1661603. 40969606 10.3389/fnut.2025.1661603 PMC12441046

[20] Ogresta D Mrzljak A Cigrovski Berkovic M Bilic-Curcic I Stojsavljevic-Shapeski S Virovic-Jukic L. Coagulation and endothelial dysfunction associated with NAFLD: Current status and therapeutic implications. J Clin Transl Hepatol. 2022;10:339–55. 35528987 10.14218/JCTH.2021.00268 PMC9039716

[21] Gallo G Nalli G Baratta F Desideri G Savoia C. Metabolic dysfunction-associated steatotic liver disease: a silent driver of cardiovascular risk and a new target for intervention. Int J Mol Sci . 2025;26:8081. 40869400 10.3390/ijms26168081 PMC12386202

[22] Bagot CN Arya R. Virchow and his triad: a question of attribution. Br J Haematol. 2008;143:180–90. 18783400 10.1111/j.1365-2141.2008.07323.x

[23] Azar J Rao A Oropallo A. Chronic venous insufficiency: a comprehensive review of management. J Wound Care. 2022;31:510–9. 35678787 10.12968/jowc.2022.31.6.510

[24] Rinde LB Småbrekke B Mathiesen EB. Ischemic stroke and risk of venous thromboembolism in the general population: the tromsø study. J Am Heart Assoc. 2016;5:e004311. 27821402 10.1161/JAHA.116.004311 PMC5210332

[25] Morelli VM Sejrup JK Småbrekke B. The role of stroke as a trigger for incident venous thromboembolism: results from a population-based case-crossover study. TH Open. 2019;3:e50–7. 31249982 10.1055/s-0039-1681020 PMC6524907

[26] Engbers MJ van Hylckama Vlieg A Rosendaal FR. Venous thrombosis in the elderly: incidence, risk factors and risk groups. J Thromb Haemost. 2010;8:2105–12. 20629943 10.1111/j.1538-7836.2010.03986.x

[27] Yu J Li J Fu L. Anemia increases the risk of venous thromboembolism? Insights from genome-wide association studies. Hematology. 2025;30:2555039. 40963252 10.1080/16078454.2025.2555039

[28] Byrnes JR Wolberg AS. Red blood cells in thrombosis. Blood. 2017;130:1795–9. 28811305 10.1182/blood-2017-03-745349 PMC5649548

