# Supplementary figures and images for: Construction and validation of a machine learning–based risk prediction model for venous thromboembolism in older adult patients: A multifactorial analysis of 28,231 cases
Source: Medicine (Baltimore). 2026 Jul 17;105(29):e49729. doi: 10.1097/MD.0000000000049729 (PMC13384574; doi:10.1097/MD.0000000000049729)

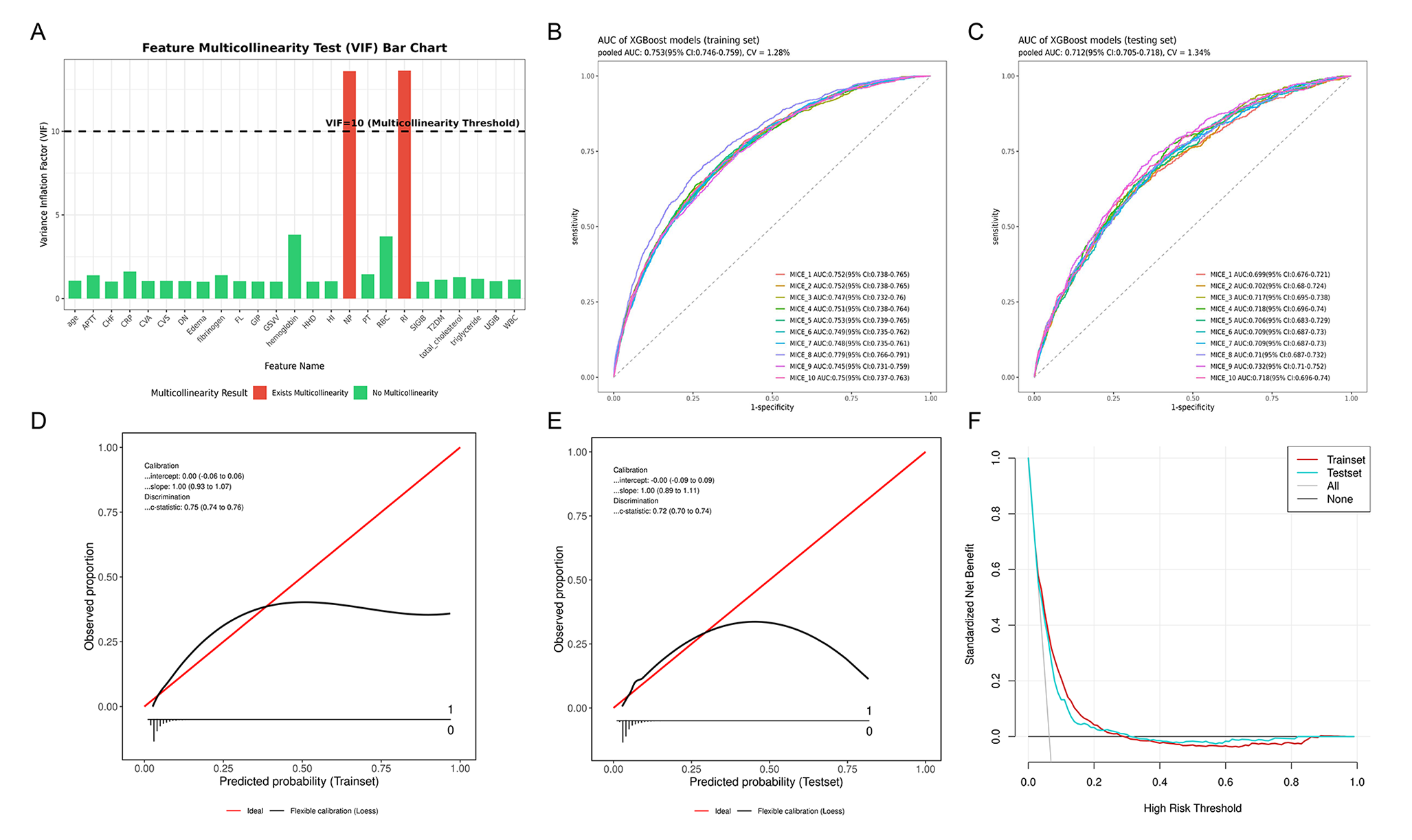

Supplement: Supplementary file 1 [file medi-105-e49729-s001.tiff]

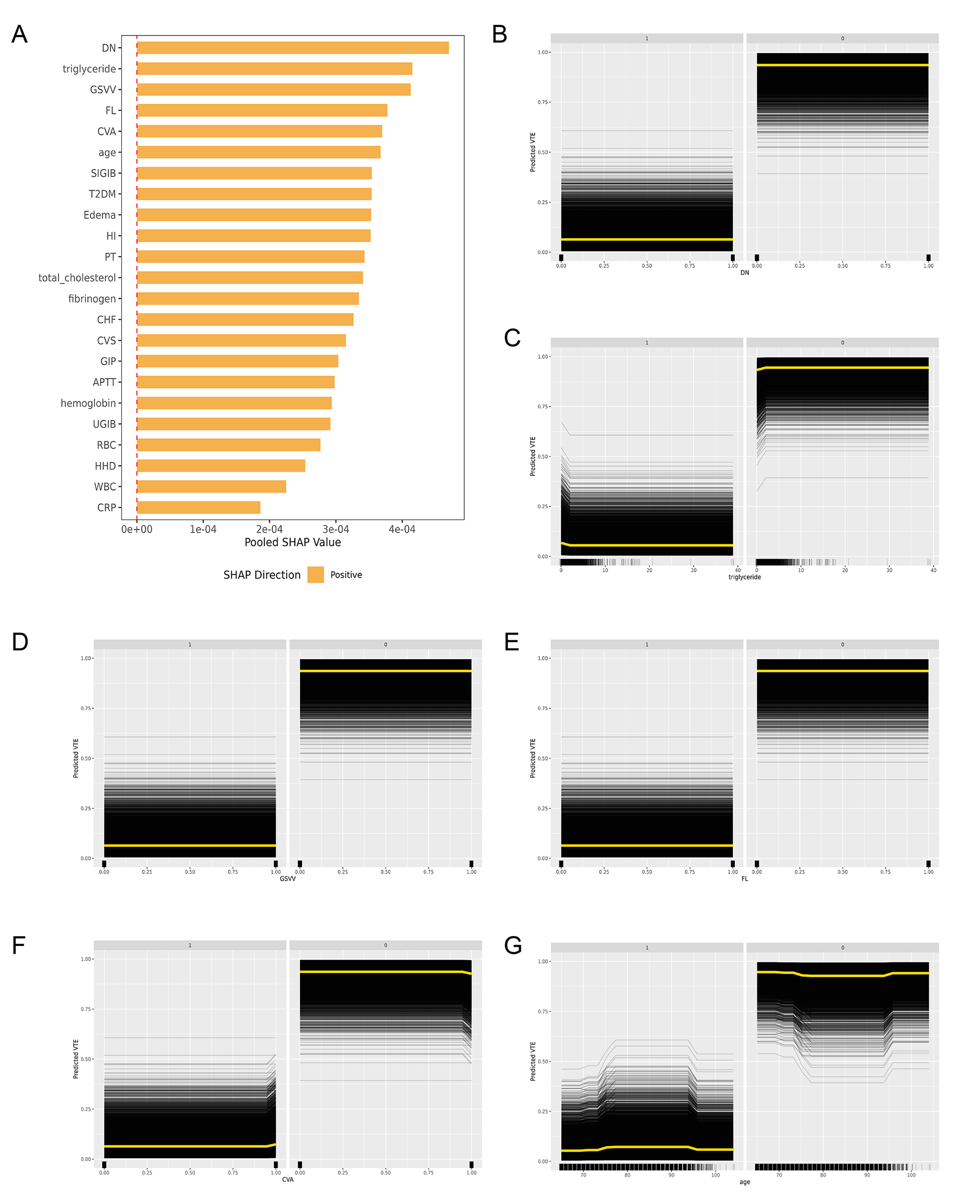

Supplement: Supplementary file 2 [file medi-105-e49729-s002.tiff]
